# Supplementary material for: Detection of Mycobacterial DNA in Human Bone Marrow
Source: Microorganisms. 2023 Jul 11;11(7):1788. doi: 10.3390/microorganisms11071788 (PMC10384717; doi:10.3390/microorganisms11071788)
Supplement: Supplementary file 1 [file microorganisms-11-01788-s001.zip › microorganisms-2488785-supplementary.pdf]

Klebsiella pneumoniae
IS*Kpn1*

```
451  gctggaaaaa  ctctcccccg  aagagctcag  ggccgaactg  cgttatctgc
                                          ISKpn1 Fw  →
501  gggcagagaa  tgcctatcta  aaaaagttga  aagccttggt  tcagagcgaa
551  aaaaatggca  aaaagccctg  ataatcagtg  aactaaggca  tgaacacgct
601  ctgcgggacc  ttctgcgggc  ggccggtatg  tcccgtagca  cgtggtatta
651  cattatgaat  gcactgaagc  aaggggacag  gtatgcgggt  cttaaagaga
701  acatcaggaa  gatataccac  tatcacaaag  gtcgtatggc  taccgcagga
                                                ←  ISKpn1 Rev
751  tcacgctcgc  actaagaaaa  caggggctgc  ggataaacca  taaaacagtg
801  cagcggctga  tggcagaact  gtcactccgg  tctgtgataa  gggcgaaaaa
```

Salmonella enterica
IS*200*

```
231  acatagtttt  cgcgcccaaa  taccgaagac  attaagaaaa  ttgtgtgaat
281  ggaaaaacgt  acgaattctg  gaagcagaat  gttgtgcata  tcatattcac
331  atgcttctgg  agatcccgcc  gaagatgagt  gtgtcgagtt  tcatgggata
                               IS200 Fw  →
381  tctgaagggt  aaaagtagtc  tgatgcttta  cgagcagttt  ggggatctaa
431  aattcaaata  caggaacagg  gagttctggt  gcagagggta  ctatgtcgat
                                        ←  IS200 Rev
481  acggtgggta  agaacacggc  gaagatacag  gactacataa  agcacgagct
531  tgaagaggat  aaaatgggtg  agcaattatc  catcccctat  ccgggcagcc
```
